# Analysis of Elemental Concentrations and Risk Assessment of Prepared Livestock and Poultry Meat Dishes Sold in Zhejiang Province

**DOI:** 10.3390/foods15010073

**Published:** 2025-12-25

**Authors:** Chenyang Zheng, Ying Tan, Zhengyan Hu, Jingshun Zhang, Jun Tang

**Affiliations:** 1 Zhejiang Provincial Center for Disease Control and Prevention, Hangzhou 310051, China; cyzheng@cdc.zj.cn (C.Z.); yint@cdc.zj.cn (Y.T.); zhyhu@cdc.zj.cn (Z.H.); jshzhang@cdc.zj.cn (J.Z.); 2 NHC Specialty Laboratory of Food Safety Risk Assessment and Standard Development, Hangzhou 310051, China

**Keywords:** ICP-MS, prepared livestock and poultry dishes, elemental exposure, health risks

## Abstract

This study assessed elemental exposure and health risks in 35 prepared livestock and poultry dishes from Zhejiang Province using inductively coupled plasma mass spectrometry (ICP-MS). Aluminum (Al) showed the highest concentrations, while strontium (Sr) and barium (Ba) were moderate; other elements (molybdenum (Mo), chromium (Cr), lead (Pb), cadmium (Cd), thallium (Tl), cobalt (Co), arsenic (As)) were low; and nickel (Ni) was undetected. All dishes complied with GB 2762-2022 limits. Although seven dishes showed mild-to-moderate single-factor contamination, Nemerow indices confirmed safe levels (P_c_ < 0.7). Principal Component Analysis (PCA) and Hierarchical Cluster Analysis (HCA) indicated that processing methods drove contamination profiles, with deep-fried products accumulating higher metal levels than stir-fried or boiled ones. While non-carcinogenic risks were acceptable for adults, children showed higher susceptibility with Total Target Hazard Quotients (TTHQ) values nearly double those of adults, exceeding safety thresholds in certain dishes primarily due to As and Cr. Carcinogenic risks for hexavalent chromium (Cr(VI)) and inorganic arsenic (iAs) were acceptable (1 × 10^−6^ to 1 × 10^−4^) for 32 dishes. After speciation-based recalibration, the remaining three dishes also fell within safe limits. Overall, exposure risks are low, though specific deep-fried products warrant monitoring.

## 1. Introduction

Prepared dishes have experienced rapid market growth driven by urbanization and changing dietary patterns, with China’s market reaching 516.5 billion yuan in 2023 [1,2]. However, quality and safety concerns have emerged alongside this expansion. In 2024, China established production standards and formal definitions for prepared dishes [3], while the United States and the United Kingdom issued microbiological safety guidelines for prepared foods [4,5], signaling heightened regulatory attention to this emerging category. Despite this regulatory focus, specific standards for monitoring chemical contaminants remain notably absent. Furthermore, comprehensive data on trace element contamination patterns and quantitative health risk assessments for prepared dishes are lacking in the scientific literature, representing a critical knowledge gap in food safety research.

Among the diverse categories of prepared dishes, livestock and poultry products warrant particular attention due to their substantial market share and unique contamination vulnerabilities [6]. In 2022, livestock and poultry-based prepared dishes comprised 29% of China’s prepared food market, representing the largest single category and thus a major dietary exposure pathway for consumers [7]. Toxic elements including lead (Pb), arsenic (As) and inorganic arsenic (iAs), mercury (Hg), cadmium (Cd), hexavalent chromium (Cr(VI)) and nickel (Ni) are classified as human carcinogens by the International Agency for Research on Cancer (IARC) and represent critical food safety indicators [8]. Livestock and poultry tissues exhibit substantially higher bioaccumulation capacity for these elements compared to plant-based foods, Cd levels in pig kidney reach 1.7 mg kg^−1^ (17 fold higher than spinach) [9,10], and Pb in bovine liver can exceed 81 mg kg^−1^ (300–650 fold higher than cereals) [11,12]. The vulnerability of prepared livestock and poultry dishes to elemental contamination is particularly concerning because contamination risks are compounded at multiple stages. Inherent biological accumulation in animal tissues is augmented by processing-introduced contamination through thermal treatment and additives [13], and further exacerbated by potential metal migration from packaging materials [14], with transfer rates from food-contact polymers reaching 8.38% for zinc (Zn) [15]. However, despite these multiple contamination pathways, systematic research on multi-element contamination in prepared dishes remains limited. Existing studies on trace elements in meat products typically analyze fresh or minimally processed samples [9,11,16,17], fundamentally failing to capture the cumulative contamination effects of preparation, packaging, and storage that define prepared dishes.

This study systematically characterized multi-element contamination and quantified health risks in commercial prepared livestock and poultry dishes. Using validated inductively coupled plasma mass spectrometry (ICP-MS) methodology [18], eleven elements (aluminum (Al), Cr, cobalt (Co), Ni, strontium (Sr), molybdenum (Mo), barium (Ba), Cd, thallium (Tl), Pb, As) were analyzed in thirty-five prepared dishes from Zhejiang Province. We encompassed both regulated priority metals and emerging contaminants overlooked in previous meat studies [16,17], and conducted differentiated health risk assessments for adults versus children using actual commercial product data [9,11]. This integrated approach establishes the baseline dataset on elemental safety in prepared livestock and poultry dishes and provides evidence-based risk information to protect consumers in China’s rapidly expanding prepared food sector.

## 2. Materials and Methods

### 2.1. Instruments and Reagents

NexION 300D ICP-MS, featuring a concentric nebulizer and a collision reaction cell (PerkinElmer, Waltham, MA, USA); Mars 6 microwave digestion system with high-pressure polytetrafluoroethylene digestion vessels (CEM, Matthews, NC, USA); AL 204 analytical balance with a precision of 0.0001 g (Mettler Toledo, Columbus, OH, USA); LB20ES tissue homogenizer (Waring, Torrington, CT, USA); Milli-Q automatic ultrapure water system (Millipore, Burlington, MA, USA). Mixed standard solution containing Cr, Co, Ni, Sr, Mo, Ba, Cd, Tl, Pb, As, and Al elements at 100 μg mL^−1^ (Agilent, Santa Clara, CA, USA); mixed internal standard solution containing scandium (Sc), germanium (Ge), yttrium (Y), indium (In), and bismuth (Bi) at 10 μg mL^−1^ (AccuTrace Reference Standard, New Haven, CT, USA); tuning solution containing lithium (Li), Y, cerium (Ce), Tl, and Co elements at 1 ng mL^−1^ (PerkinElmer, Waltham, MA, USA). Bovine liver standard reference material SRM1577c (National Institute of Standards and Technology, Gaithersburg, MD, USA); chicken meat standard reference material GBW10018 (National Institute of Metrology, Beijing, China). Nitric acid of superior grade purity (Merck, Darmstadt, Germany). Ultrapure water was used throughout the experiment.

### 2.2. Sample Pretreatment

Thirty-five prepared livestock and poultry meat dishes were purchased in November 2024 from supermarkets or chain retail outlets in three representative cities of Zhejiang Province, namely Hangzhou, Ningbo and Wenzhou. In each city, three supermarkets or chain stores located in different urban areas were selected, and for each type of prepared meat dish one retail package was purchased from each store. The 35 dishes were classified into three categories: eleven meat cutlets and patties, including chicken steak, beef steak, pork steak, chicken patty, fish patty and similar items from seven manufacturers; seven fried meat and sausage products, including chicken nuggets, crispy meat, roasted sausage and similar items from five manufacturers; and seventeen stir-fried or boiled meat products, including sweet and sour meat, boiled meat, braised meat and similar items from eleven manufacturers. For each dish type, the edible portions of the nine retail packages were removed, combined and homogenized to obtain a single composite sample. Approximately 0.5–1.0 g of the homogenized sample was accurately weighed (precision to 0.0001 g) into a polytetrafluoroethylene digestion vessel, and 5 mL of nitric acid was added. The mixture was allowed to stand at low temperature for 1–2 h. The samples were then digested in a microwave digestion system with the following program: step one, ramp to 100 °C in 40 min and hold for 20 min; step two, ramp to 140 °C in 15 min and hold for 15 min; step three, ramp to 195 °C in 20 min and hold for 10 min. After digestion and cooling to room temperature, the digest was diluted to 20 mL. A reagent blank was prepared simultaneously.

### 2.3. ICP-MS Parameters

The ICP-MS was equipped with a collision reaction cell using helium as the collision gas. Instrument conditions were optimized with a tuning solution at 1 ng mL^−1^ to ensure that sensitivity, oxide levels, doubly charged ion ratios, and resolution met analytical requirements. The optimal ICP-MS operating parameters obtained are shown in Table 1.

### 2.4. Quality Control

The method was validated in terms of linearity, limits of detection (LOD), limits of quantification (LOQ), accuracy, and repeatability. An appropriate amount of mixed multi element standard solution was diluted with 1% nitric acid to prepare mixed calibration standards of Cr, Co, Ni, As, Sr, Mo, Cd, Ba, Tl, and Pb at concentrations of 0, 1.0, 2.0, 4.0, 6.0, 8.0, 10.0, 20.0, and 50.0 µg L^−1^. Al standard solutions were prepared at 10.0, 20.0, 50.0, 60.0, 80.0, 100.0, 200.0, and 500.0 µg L^−1^. Calibration curves were established, and the correlation coefficients (r) were required to be at least 0.999. According to the principle that the mass number of the internal standard isotope should be close to that of the analyte, Sc, Ge, Y, In, and Bi were selected as internal standards. In previous studies, LOD and LOQ are calculated using the standard deviation of the response and the slope of the calibration curve [19]. In this study, LOD and LOQ were defined as the concentrations corresponding to a response equivalent to three times or ten times the range of the baseline signal of the matrix blank [20]. According to the handling principles for non detected data recommended in the second meeting of the WHO Global Environment Monitoring System and Food Contamination Monitoring and Assessment Programme (GEMS/FOOD) on the “credibility assessment of low level contaminants in food” [21], non detected results in this evaluation were assigned one half of the LOD for statistical analysis. The accuracy of the method was verified by determining 11 elements in SRM1577c and GBW10018. Repeatability was evaluated by analyzing each standard reference material six times within the same day and calculating the relative standard deviation (RSD).

### 2.5. Multivariate Statistical Analysis

To elucidate the contamination patterns and relationships among different prepared dishes, Principal Component Analysis (PCA) and Hierarchical Cluster Analysis (HCA) were performed. Prior to analysis, the elemental concentration data were log-transformed to improve normality and reduce the influence of extreme values. PCA was employed to identify the principal components (PCs) contributing to the variance in elemental distribution and to visualize the clustering of samples based on their contamination profiles. HCA was conducted to group the dishes into clusters with similar elemental characteristics. The squared Euclidean distance was selected as the similarity measure, and Ward’s method was applied as the linkage criterion to minimize the within-cluster variance.

### 2.6. Risk Assessment Methods

The pollution index method was used to evaluate contamination levels of hazardous elements in prepared livestock and poultry meat products [22], following Formulas (1) and (2).

(1) $$P_{i}=C_{i}/S_{i}$$

(2) $$P_{c}=\sqrt{\left({P_{ave}}^{2}+{P_{max}}^{2}\right)/2}$$

In Formula (1), P_i_ represents the single-factor pollution index of hazardous element i. C_i_ is the concentration of element i, expressed in mg kg^−1^. S_i_ is the maximum limit standard value for element i, expressed in mg kg^−1^. Since national standards for contaminants in prepared dishes have not yet been issued, the limit values for Pb, Cd, Cr, and As in meat and meat products specified in GB 2762-2022 were used as references [23], as shown in Table 2. Classification based on P_i_ values was as follows: P_i_ < 0.2 indicates no pollution, 0.2 ≤ P_i_ < 0.6 indicates slight pollution, 0.6 ≤ P_i_ < 1.0 indicates moderate pollution, and P_i_ ≥ 1.0 indicates severe pollution.

In Formula (2), P_c_ represents the Nemerow composite pollution index, P_ave_ is the mean value of the single-factor pollution indices, and P_max_ is the maximum single-factor pollution index. Classification based on P_c_ values is as follows: P_c_ ≤ 0.7 indicates a safe level, 0.7 < P_c_ ≤ 1.0 indicates a warning level, 1.0 < P_c_ ≤ 2.0 indicates slight pollution, 2.0 < P_c_ ≤ 3.0 indicates moderate pollution, and P_c_ > 3.0 indicates severe pollution.

### 2.7. Health Risk Assessment of Hazardous Elements

(1) Non-carcinogenic risk assessment

The United States Environmental Protection Agency (EPA) proposed the Target Hazard Quotients (THQ) method for estimating the potential non carcinogenic risks associated with long term exposure to contaminants in food. The non carcinogenic risks posed by single hazardous elements and by multiple hazardous elements in prepared livestock and poultry meat products were evaluated by calculating the THQ and the Total Target Hazard Quotients (TTHQ), according to Formulas (3) and (4).

(3) $$THQ=\frac{EDI}{RfD_{o}}=\frac{C\times EF\times ED\times IR\times CF}{RfD_{o}\times BW\times AT}$$

(4) $$TTHQ=\sum_{i=1}^{n}THQ_{i}$$

In Formula (3), EDI is the estimated daily intake. C is the concentration of the hazardous element, mg kg^−1^. EF is the exposure frequency, 365 d yr^−1^. ED is the exposure duration, 70 year. IR is the daily intake, and the value used in this study was the average daily consumption of livestock and poultry meat including pork, beef and mutton, and poultry by residents of Zhejiang Province in 2024, which was 150.6 g d^−1^ [24]. CF is the conversion factor, 10^−3^ kg g^−1^. RfD_o_ is the oral reference dose for non carcinogenic effects. The values for Pb, Cd, Cr, and As were 0.0035, 0.001, 0.003, and 0.0003 mg kg^−1^ d^−1^, respectively [25]. BW values were 61.8 kg for adults and 30.0 kg for children [9,26]. AT is the average exposure time, 25,550 day. When THQ or TTHQ < 1.0, the health risk posed by the hazardous element is considered acceptable. A greater THQ or TTHQ value indicates a higher potential health risk [27].

(2) Carcinogenic risk assessment

Carcinogenic risk (R) was evaluated using the carcinogenic slope factor (SF) recommended by the EPA, and was calculated according to Formula (5).

(5) $$R=CDI\times SF_{o}=\frac{C\times EF\times ED\times IR\times CF\times SF_{o}}{BW\times AT}$$

Chronic Daily Intake (CDI) represents the chronic daily intake. SF_o_ is the oral carcinogenic slope factor. The SF_o_ values for iAs and Cr(VI) are 1.5 and 0.16 mg (kg·d)^−1^, respectively [28,29]. R between 1 × 10^−6^ to 1 × 10^−4^ is considered acceptable. A higher R value indicates a higher carcinogenic risk [30].

### 2.8. Data Processing

Microsoft Excel 2016 was used for initial data processing. Since the data for the prepared dishes did not follow a normal distribution, the results were expressed as the median and interquartile (IQR = Q3 − Q1). Outliers were defined as values (<Q1 − 1.5 × IQR or >Q3 + 1.5 × IQR). PCA and HCA were performed using OriginPro 2021 to visualize the distribution patterns and similarities among samples. For HCA, the Euclidean distance was used as the similarity measure, and Ward’s method was selected for linkage. Elements undetected in all samples were excluded from the analysis to ensure model robustness.

## 3. Results and Discussion

### 3.1. Linear Range and LOD

Under the ICP-MS parameters described in Section 2.3, 11 target elements were analyzed. Using the internal standard corrected signal intensity as the ordinate and the concentrations of the standard curve solutions described in Section 2.4 as the abscissa, linear regression equations and r within each concentration range were obtained. The r of all calibration curves were greater than 0.999, indicating excellent linearity within the tested ranges. When the sample mass was 0.5 g and the digestion volume was 20 mL, the LOD and LOQ for the 11 elements are shown in Table 3.

### 3.2. Accuracy and Precision

To verify the accuracy and precision of the method, two certified standard reference materials, SRM1577c and GBW10018, were selected. Six parallel preparations were made for each standard reference material, and two portions of each were inserted after every ten samples during analysis. Their measured values were used to determine whether any systematic error occurred. The results are shown in Table 4. For both standard reference materials, the measured concentrations of all elements fell within the certified ranges, and the RSD were between 1.57% and 15.1%. These results indicate that the method has good precision and reproducibility, and that the analytical results obtained for samples in the same batch under the above conditions can be considered valid.

### 3.3. Multi-Element Concentrations in Prepared Livestock and Poultry Meat Products

The concentrations of 11 elements in 35 prepared livestock and poultry meat dishes were determined, and the descriptive statistical results for each element are shown in Table 5. The results indicated that Al exhibited relatively high concentrations greater than 1.0 mg kg^−1^, with an IQR of 3.88 mg kg^−1^. Previous studies also reported that Al was the most abundant metal detected in chicken and liver samples from Egypt and conducted corresponding health risk assessments [16]. To evaluate Al exposure, we compared our intake estimates using the EDI term embedded in Formula (3) with the Provisional Tolerable Weekly Intake (PTWI) for Al set by the Joint FAO/WHO Expert Committee on Food Additives (JECFA) of Al (2 mg kg bw^−1^ w^−1^) [31]; at the maximum observed Al concentration of 22.4 mg kg^−1^, EDI and EWI were 0.0546 mg kg bw^−1^ d^−1^ and 0.382 mg kg bw^−1^ w^−1^, respectively, corresponding to 19.1% of the PTWI. Sr and Ba were present at moderate levels greater than 0.1 mg kg^−1^, with some Sr concentrations exceeding 2.0 mg kg^−1^. Although Ba and Sr are not currently regulated in meat products, emerging evidence of their occurrence in animal-derived and composite foods, along with potential risks from chronic exposure in sensitive populations, underscores the need for precautionary attention and further toxicological and surveillance research [32,33]. Mo, Cr, and Pb were present at relatively low concentrations, with most values ranging from 0.01 to 0.1 mg kg^−1^. Cd, Tl, Co, and As occurred at extremely low concentrations, with most values below 0.01 mg kg^−1^. Ni was not detected in any sample, which is consistent with literature indicating that meat is not a major contributor to dietary nickel exposure [34]. The detection rates of Al, Ba, Sr, Co, Mo, As, Pb, Cr, Tl, Cd, and Ni, from highest to lowest, were 100%, 100%, 97.1%, 94.3%, 91.4%, 88.6%, 48.6%, 48.6%, 42.9%, 25.7%, and 0%, respectively. Regarding outliers, no outliers were observed for Ba and Ni. Al showed one outlier. Co, As, and Sr each had two outliers. Cr, Mo, and Tl each had three outliers, while Pb and Cd each had four outliers. According to GB 2762-2022 limits for As, Pb, Cr, and Cd in meat and meat products, all samples complied with national standards. The high-value outlier for As occurred in the codfish cake (containing pork loin), with a concentration of 0.47 mg kg^−1^, approaching the limit of 0.5 mg kg^−1^. Pb outliers were observed in quinoa chicken steak, black pepper chicken pieces, vegetable chicken patty, and sweet and sour pork. Cr outliers were found in pork tripe chicken and marinated meat sauce products. Cd outliers were detected in scallion pork, vegetable chicken patty, codfish cake (containing pork loin), and marinated meat sauce. At the individual dish level, the sweet and sour pork dish contained two high outliers of Pb 0.167 mg kg^−1^ and Al 22.4 mg kg^−1^. Previous research has suggested that acidic sweet and sour systems may enhance the migration of Pb and Al sources or may relate to the use of Al-containing leavening agents [15]. The vegetable chicken patty dish contained six outliers for Co, Mo, Tl, Pb, Cr, and Cd, among which Co 0.030 mg kg^−1^, Mo 0.44 mg kg^−1^, Tl 0.002 mg kg^−1^, and Cd 0.020 mg kg^−1^ were markedly above the upper quartile. The composite formulation containing vegetables and spices may be more prone to introducing trace metals [10], consistent with recent reports on background levels and variability of Cd and Pb in plant-based ingredients [10,27].

### 3.4. Multivariate Statistical Analysis

#### 3.4.1. PCA of Contamination Patterns

**Figure 1:**
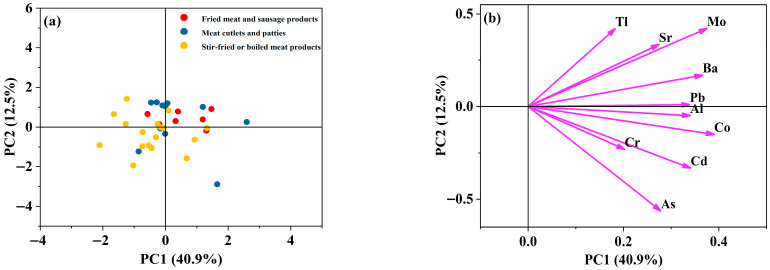
Score plot (**a**) and loading plot (**b**) of PCA for multi-element profiles in prepared livestock and poultry meat dishes.

PCA of log-transformed elemental concentration data revealed a complex contamination landscape characterized by two dominant axes of variation that collectively explained 53.4% of total variance, as shown in Figure 1a. PC1, explaining the largest proportion of variance, showed strong positive loadings for Co (0.390), Mo (0.375), Ba (0.366), Al (0.340), Cd (0.341), Pb (0.339), As (0.278), and Sr (0.274) (see Figure 1b). This component primarily represents overall heavy metal contamination levels and appears to be associated with processing intensity. Most fried meat and sausage products exhibited positive PC1 scores ranging from −0.57 to 1.47, suggesting elevated metal accumulation during high-temperature frying processes. This finding aligns with previous studies demonstrating that deep-frying can concentrate metals through moisture loss and fat oxidation [35]. Meat cutlets and patties displayed the widest distribution along PC1, ranging from −0.86 to 2.59, with vegetable chicken patty showing exceptionally high contamination at PC1 = 2.59, possibly reflecting variability in raw material or processing conditions. Stir-fried or boiled products predominantly exhibited negative to low positive PC1 scores, ranging from −2.10 to 1.32, indicating that gentler cooking methods result in lower overall metal retention. This is consistent with previous studies reporting significant metal leaching during boiling [36]. PC2 was characterized by high positive loading for Tl (0.420) and strong negative loading for As (−0.563) (see Figure 1b), suggesting these elements originate from independent contamination sources or behave differently during processing. The inverse relationship between Tl and As has been previously observed in food processing environments and may reflect different volatilization behaviors at cooking temperatures [37]. Meat cutlets and patties showed the greatest variability along PC2, particularly codfish cake (containing pork loin) with PC2 = −2.89, indicating distinct As enrichment patterns in certain cutlet formulations.

#### 3.4.2. HCA of Clustering Patterns

**Figure 2:**
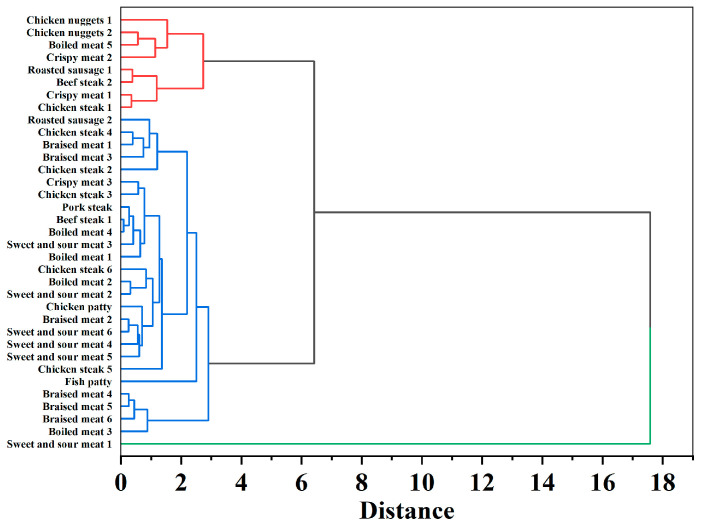
HCA dendrogram of elemental profiles in prepared livestock and poultry meat dishes.

To further examine relationships among samples, HCA was performed using Euclidean distance and Ward’s linkage. The dendrogram in Figure 2 shows three distinct clusters that correspond to the processing-related patterns observed in PCA. Cluster 1 (Red Branch) forms a tight grouping at a linkage distance below 3.0, encompassing the majority of deep-fried and heavily processed items, such as Chicken Nuggets, Roasted Sausage, Crispy Meat, and Meat Steak. This aligns with positive PC1 scores, reflecting high Al, Pb, and Cd contamination from industrial processing equipment and high-temperature frying [38]. Cluster 2 (Blue Branch), forming at a linkage distance slightly above 3.0, primarily encompasses whole meat cutlets and patties, and stir-fried or boiled meat products, corresponding to negative to low positive PC1 scores. While wet cooking promotes metal leaching in stir-fried or boiled meats [36], the low retention in cutlets and patties is attributed to lean muscle usage and ingredient dilution [39]. The Fish Patty forms a distinct sub-branch due to high As concentration of 0.47 mg kg^−1^, characteristic of marine based ingredients. Cluster 3 (Green Branch) shows Sweet and Sour Meat as a significant outlier at linkage distance of 17.6, with exceptionally high Al concentration of 22.4 mg kg^−1^. This likely reflects Al containing food additives or migration from cookware during acidic sauce preparation [15]. HCA reinforces PCA findings, showing that processing method primarily determines trace element profiles while isolating contamination outliers.

### 3.5. Assessment of Hazardous Element Contamination

Using the maximum limit values corresponding to each food category in Table 2 and the measured concentrations of Pb, Cd, Cr, and As in the 35 prepared livestock and poultry meat dishes, the single-factor pollution index and the Nemerow composite pollution index were calculated according to Equations (1) and (2) (Table 6). Among these dishes, 28 had a single-factor pollution index (P_i_) < 0.2, indicating an unpolluted level; their Nemerow composite pollution index (P_c_) values were all < 0.7, corresponding to a safe level. The P_c_ values followed the order: fried meat and sausage products > stir-fried or boiled meat products > meat cutlets and patties. Seven dishes exhibited P_i_ ≥ 0.2. Specifically, black pepper chicken nuggets, quinoa chicken steak, vegetable chicken patty, and sweet and sour pork were classified as having slight Pb pollution; braised meat paste and pork-tripe chicken exhibited slight and moderate Cr pollution, respectively; and the codfish cake (containing pork loin) showed moderate As pollution. Despite these elevated single-factor indices, all seven samples had P_c_ values within the safe level. Previous studies have reported substantial spatial and batch-to-batch variability of hazardous elements such as As, Pb, Cd, and Cr in meat and meat products, with some individual samples exceeding maximum limits while the overall risk remains acceptable [40]. This aligns with the findings of the present study.

### 3.6. Dietary Exposure Risk Assessment of Hazardous Elements

#### 3.6.1. Non-Carcinogenic Risk Assessment

The THQ and TTHQ of the 35 dishes were calculated according to Equations (3) and (4), and the results are presented in Table 7. Among them, 34 dishes had TTHQ values < 1, indicating no non-carcinogenic risk associated with Pb, Cr, Cd, or As in the prepared dishes. The TTHQ values followed the order: stir-fried or boiled meat products > meat cutlets and patties > fried meat and sausage products. The contribution of Cr-related THQ to the TTHQ was 92.6%, 62.1%, and 47.8% in these three categories, respectively, making Cr the predominant contributor to non-carcinogenic risk. The codfish cake (containing pork loin) had a TTHQ of 3.9, with As accounting for 97.4% of the total, suggesting a notable non-carcinogenic risk. In this study, the non-carcinogenic risk indicator was derived based on intake levels; however, average consumption, body weight, exposure frequency, and exposure duration vary considerably among populations. Children, in particular, are more sensitive to the same consumption level due to their lower body weight [41]. THQ and TTHQ values for children were approximately twice those for adults. Of particular concern, the TTHQ for children in Codfish cake (containing pork loin) reached a critical 8.0 (driven by As), while Pork-tripe chicken also exceeded the safe threshold. These results highlight the urgent need to monitor dietary exposure in children, given their higher physiological susceptibility to heavy metal accumulation.

#### 3.6.2. Carcinogenic Risk Assessment

The R of Cr(VI) and iAs for the 35 dishes were calculated using Equation (5), and the results are shown in Table 8. Among them, 32 dishes had R values within the range of 1 × 10^−6^ to 1 × 10^−4^, which is considered acceptable. No significant differences in R values for Cr(VI) or iAs were observed among the three categories of prepared meat products. Three dishes had R values exceeding 1 × 10^−4^: the Cr(VI)-related risks of the braised meat paste and pork-tripe chicken were 1.0 × 10^−4^ and 2.4 × 10^−4^, respectively, while the iAs-related risk of the codfish cake (containing pork loin) was 1.7 × 10^−3^, indicating a certain carcinogenic risk. In common fish species, the proportion of iAs typically accounts for less than 2% of total As [42]. Thus, treating total As as 100% iAs leads to a substantial overestimation of carcinogenic risk. After recalculation using literature-based iAs proportions, the R value for the codfish cake (containing pork loin) decreased to an acceptable level (3.4 × 10^−5^). Similarly, Cr(VI) in foods can be fully reduced to Cr(III) during high-temperature or acidic cooking processes [43]. Therefore, equating total Cr with Cr(VI) also generates exposure estimates far above realistic levels. In future work, speciation analysis will be performed on samples identified as having elevated risk or abnormal total concentrations, to substantially reduce assessment uncertainty and provide more reliable quantitative evidence for risk management.

## 4. Conclusions

In this study, eleven elements in 35 commercially available prepared livestock and poultry meat dishes from Zhejiang Province were quantified using ICP-MS. Al was the predominant element, followed by moderate levels of Sr and Ba, while toxic heavy metals (Pb, Cd, Cr, As) were generally low, and Ni was undetected. All dishes complied with the regulatory limits of GB 2762-2022. Although single-factor indices flagged mild contamination in seven dishes, the Nemerow comprehensive pollution index (P_c_ < 0.7) confirmed that overall contamination levels remained low across all product categories. Multivariate analysis highlighted that processing techniques significantly influence elemental distribution; specifically, high-temperature frying was associated with elevated concentrations of Al, Pb, and Cd, whereas boiling or stir-frying resulted in lower retention of these contaminants. Age-stratified analysis revealed that children face significantly higher cumulative heavy metal risks than adults, with TTHQ values exceeding safety limits in specific dishes despite being safe for the adult population. For carcinogenic risks, while three dishes initially exceeded safe thresholds for Cr(VI) or iAs, recalibration using literature-based speciation ratios reduced these risks to acceptable levels. This finding underscores that risk assessments based solely on total element concentrations may be overly conservative and highlights the critical role of speciation analysis in reducing uncertainty.

However, limitations remain, particularly the reliance on literature-based ratios rather than direct speciation, and the lack of harmonized acute oral reference values. Future studies should integrate direct speciation analysis and acute risk endpoints to refine exposure assessments. Overall, this study provides essential data to support regulatory oversight and process optimization in the prepared food industry, facilitating more accurate consumer health protection, especially for vulnerable populations such as children.

## Figures and Tables

**Table 1:** Instrumental operating conditions.

ICP-MS Parameters	
RF power	1300 W
Nebulier flow rate	1.05 L min^−1^
Plasma gas flow rate	18.0 L min^−1^
Auxiliary gas flow rate	1.2 L min^−1^
Cell gas B flow rate	1.4 mL min^−1^ 5% H_2_ in He
RPQ	0.45
Sampling cone/ Skimmer cone	1.0/0.4 (Pt)
Isotopes monitored	^27^Al, ^52^Cr, ^59^Co, ^60^Ni, ^75^As, ^88^Sr, ^95^Mo, ^111^Cd, ^137^Ba, ^205^Tl, ^208^Pb, ^45^Sc, ^73^Ge, ^89^Y, ^115^In, ^209^Bi
Dwell time	50 ms
Sweeps	40
Data acquisition mode	Time resolved analysis

**Table 2:** Maximum Levels (MLs) for meat and meat products.

Elements	Food Category	ML (mg kg^−1^)
Pb	Meat products (excluding livestock and poultry offal products)	0.3
	Livestock and poultry offal products	0.5
Cd	Meat and meat products (excluding livestock and poultry offal and their products)	0.1
	Livestock and poultry liver and liver products	0.5
	Livestock and poultry kidney and kidney products	1.0
Cr	Meat and meat products	1.0
As	Meat and meat products	0.5

**Table 3:** Linear equations and the limits of detection.

Elements	Linear Equation	Linear Range (μg L^−1^)	Correlation Coefficient (r)	LOD (mg kg^−1^)	LOQ (mg kg^−1^)
Al	y = 301.97x + 736.63	10.0–500.0	0.9999	0.50	1.67
Cr	y = 11,777.62x + 604.81	1.0–50.0	1.0000	0.051	0.17
Co	y = 32,024.79x + 38.37	1.0–50.0	0.9999	0.0010	0.0033
Ni	y = 8008.09x + 358.12	1.0–50.0	0.9999	0.20	0.67
As	y = 1612.19x + 37.08	1.0–50.0	1.0000	0.0021	0.0070
Sr	y = 9050.98x + 111.65	1.0–50.0	1.0000	0.20	0.67
Mo	y = 10,306.13x + 10.01	1.0–50.0	0.9998	0.010	0.033
Cd	y = 5565.07x + 4.98	1.0–50.0	0.9999	0.0021	0.0071
Ba	y = 4418.21x + 148.00	1.0–50.0	0.9999	0.020	0.067
Tl	y = 173,927.43x + 141.66	1.0–50.0	0.9998	0.00018	0.00060
Pb	y = 1,034,842.83x + 3458.30	1.0–50.0	0.9999	0.020	0.067

**Table 4:** Determination results of standard substances (mg kg^−1^).

Elements	SRM1577c			GBW10018		
	Certified Values	Measured Average Values	RSD (%)	Certified Values	Measured Average Values	RSD (%)
Al				160 ± 30	152	4.50
Cr	0.053 ± 0.014	0.049	15.1	0.59 ± 0.11	0.56	3.23
Co	0.300 ± 0.018	0.303	3.12	0.01	0.01	1.57
Ni	0.044 ± 0.009	0.048	6.63	0.15 ± 0.03	0.14	4.90
As	0.020 ± 0.001	0.020	9.20	0.109 ± 0.013	0.107	4.02
Sr	0.095 ± 0.004	0.097	4.29	0.64 ± 0.08	0.69	5.91
Mo	3.3 ± 0.13	3.27	1.77	0.11 ± 0.01	0.12	3.65
Cd	0.097 ± 0.001	0.096	2.12	5.0	5.0	2.32
Ba				1.5 ± 0.4	1.7	6.60
Tl				0.014	0.014	3.20
Pb	0.063 ± 0.001	0.064	5.85	0.11 ± 0.02	0.12	6.77

**Table 5:** Descriptive statistics of multi-element concentrations in prepared livestock and poultry meat dishes (mg kg^−1^).

Elements	Detection Rate (%)	Median	Q1	Q3	IQR	Outliers
Al	100	3.88	2.92	6.77	3.85	22.4
Cr	48.6	0.026	0.026	0.066	0.040	0.18; 0.29; 0.62
Co	94.3	0.003	0.002	0.006	0.004	0.021; 0.030
Ni	0.00	0.10	0.10	0.10	0.00	
As	88.6	0.003	0.002	0.005	0.003	0.012; 0.47
Sr	97.1	0.98	0.51	1.27	0.76	2.49; 2.93
Mo	91.4	0.036	0.022	0.052	0.030	0.15; 0.15; 0.44
Cd	25.7	0.001	0.001	0.0013	0.0003	0.003; 0.005; 0.013; 0.020
Ba	100	0.25	0.15	0.41	0.26	
Tl	42.9	0.0001	0.0001	0.0003	0.0002	0.002; 0.003; 0.007
Pb	48.6	0.010	0.010	0.031	0.021	0.066; 0.072; 0.084; 0.167

**Table 6:** Single-factor and Nemerow composite pollution indices in prepared livestock and poultry meat dishes.

Dishes	Pi				Pollution Level	P_c_	Safety Level
	Pb	Cd	Cr	As			
Fried meat and sausage products	0.033–0.11	0.011–0.026	0.026–0.11	0.002–0.014	Unpolluted	0.027–0.087	Safe
Stir-fried or boiled meat products	0.033–0.14	0.011–0.022	0.026–0.085	0.002–0.025	Unpolluted	0.027–0.10	Safe
Meat cutlets and patties	0.033–0.086	0.011–0.020	0.026–0.059	0.004–0.009	Unpolluted	0.027–0.065	Safe
Black pepper chicken nuggets	0.28	0.011	0.081	0.014	Slight Pb pollution	0.21	Safe
Quinoa chicken steak	0.24	0.018	0.059	0.010	Slight Pb pollution	0.18	Safe
Vegetable chicken patty	0.22	0.19	0.18	0.011	Slight Pb pollution	0.19	Safe
Sweet and sour pork	0.56	0.016	0.085	0.013	Slight Pb pollution	0.41	Safe
Braised meat paste	0.033	0.052	0.29	0.012	Slight Cr pollution	0.22	Safe
Pork-tripe chicken	0.033	0.011	0.62	0.005	Moderate Cr pollution	0.46	Safe
Codfish cake (containing pork loin)	0.10	0.13	0.026	0.94	Moderate As pollution	0.70	Safe

**Table 7:** THQ and TTHQ of heavy metals in prepared livestock and poultry meat dishes.

Dishes	Population	THQ				TTHQ	Risk Status
		Pb	Cd	Cr	As		
Fried meat and sausage products	Adults	0.007–0.058	0.003–0.006	0.021–0.086	0.009–0.057	0.039–0.18	Acceptable
	Children	0.014–0.12	0.006–0.012	0.043–0.18	0.018–0.12	0.080–0.37	Acceptable
Stir-fried or boiled meat products	Adults	0.007–0.12	0.003–0.013	0.021–0.50	0.009–0.10	0.039–0.54	Acceptable
	Children	0.014–0.25	0.006–0.027	0.043–1.03	0.018–0.21	0.080–1.11	Potential Risk (Pork-tripe chicken)
Meat cutlets and patties	Adults	0.007–0.050	0.003–0.048	0.026–0.18	0.017–3.8	0.049–3.9	Potential Risk (Codfish cake (containing pork loin))
	Children	0.014–0.10	0.006–0.099	0.054–0.37	0.035–0.093	0.10–8.0	Potential Risk (Codfish cake (containing pork loin))

**Table 8:** Carcinogenic risk in prepared livestock and poultry meat dishes.

Dishes	R		Risk Status	
	Cr(VI)	iAs	Cr(VI)	iAs
Fried meat and sausage products	1.0 × 10^−5^–4.1 × 10^−5^	4.0 × 10^−6^–2.5 × 10^−5^	Acceptable	Acceptable
Stir-fried or boiled meat products	1.0 × 10^−5^–3.3 × 10^−5^	4.0 × 10^−6^–4.5 × 10^−5^	Acceptable	Acceptable
Meat cutlets and patties	1.0 × 10^−5^–7.1 × 10^−5^	8.0 × 10^−6^–2.0 × 10^−5^	Acceptable	Acceptable
Braised meat paste	1.0 × 10^−4^	2.2 × 10^−5^	Risky	Acceptable
Pork-tripe chicken	2.4 × 10^−4^	9.0 × 10^−6^	Risky	Acceptable
Codfish cake (containing pork loin)	1.0 × 10^−5^	1.7 × 10^−3^	Acceptable	Risky

## Data Availability

The original contributions presented in the study are included in the article, further inquiries can be directed to the corresponding author.
